# Sex Differences in Predictors of Obstructive Sleep Apnea Risk Among Young Adults: A Cross-Sectional Study in Colombian University Students

**DOI:** 10.3390/jcm14196738

**Published:** 2025-09-24

**Authors:** Juan Alberto Aristizábal-Hoyos, Olga Patricia López-Soto, Héctor Fuentes-Barría, Raúl Aguilera-Eguía, Lissé Angarita-Davila, Diana Rojas-Gómez

**Affiliations:** 1 Departamento de Salud Oral, Facultad de Salud, Universidad Autónoma de Manizales, Manizales 170017, Colombia; jaristi@autonoma.edu.co (J.A.A.-H.); sonrie@autonoma.edu.co (O.P.L.-S.); 2 Vicerrectoría de Investigación e Innovación, Universidad Arturo Prat, Iquique 1110939, Chile; 3 Departamento de Salud Pública, Facultad de Medicina, Universidad Católica de la Santísima Concepción, Concepción 3349001, Chile; raguilerae@ucsc.cl; 4 Escuela de Nutrición y Dietética, Facultad de Medicina, Universidad Andres Bello, Concepción 3349001, Chile; lisse.angarita@unab.cl; 5 Escuela de Nutrición y Dietética, Facultad de Medicina, Universidad Andres Bello, Santiago 7550000, Chile; diana.rojas@unab.cl

**Keywords:** sleep apnea, obstructive, risk factors, students, anthropometry, sex characteristics

## Abstract

**Objectives**: This study aimed to identify anatomical and clinical predictors of moderate to high Obstructive Sleep Apnea (OSA) risk in a sample of university students, with an emphasis on sex-based differences. **Methods**: A cross-sectional study was conducted among 340 university students (148 males, 192 females) in Manizales, Colombia. Anthropometric measurements, anatomical features (neck circumference, Mallampati index, facial profile, molar Angle classification), and validated screening tools (STOP-BANG, Epworth Sleepiness Scale) were assessed. Multivariate logistic regression models were applied globally and stratified by sex to determine predictors of moderate/high OSA risk (STOP-BANG ≥ 3). **Results**: Males had significantly higher STOP-BANG scores, neck circumference, and prevalence of moderate/high OSA risk (23% vs. 3.1%), while females showed higher daytime sleepiness (*p* < 0.001). In the global model, neck circumference (OR = 0.57, *p* < 0.001) and Epworth score (OR = 0.86, *p* = 0.01) were significant predictors. In men, neck circumference (OR = 0.62, *p* < 0.001) and Angle’s molar classification (OR = 0.54, *p* = 0.04) were associated with risk. In women, neck circumference (OR = 0.35, *p* = 0.01) and daytime sleepiness (OR = 0.60, *p* = 0.03) remained significant. **Conclusions**: Easily accessible anatomical and clinical markers can help identify young adults at risk for OSA. Sex-specific screening approaches may enhance early detection strategies in university populations. Implementing these tools in clinical and educational settings may improve targeted prevention, facilitate timely referral to sleep specialists, and potentially reduce long-term health complications associated with undiagnosed OSA in emerging adults.

## 1. Introduction

Obstructive sleep apnea (OSA) is an underdiagnosed condition that represents a major public health concern, characterized by recurrent episodes of partial or complete upper airway obstruction during sleep [1]. These episodes lead to intermittent hypoxia, sleep fragmentation, and autonomic dysfunction, which, over time, can result in serious systemic complications. While OSA has been extensively studied in pediatric and older adult populations, a critical knowledge gap remains regarding its prevalence, risk factors, and clinical manifestations in young adults, particularly among university students [2].

Although this age group is typically perceived as healthy, they may present predisposing factors such as obesity, craniofacial anomalies, and parafunctional habits that increase susceptibility to OSA [3]. Despite these potential risks, clinical manifestations of OSA in young adults are often subtle or mistakenly attributed to lifestyle-related fatigue or academic stress. In this context, the absence of early detection and timely intervention raises the likelihood of developing hypertension, cardiovascular disease, and cognitive impairment [4,5,6]. Untreated OSA during early adulthood may initiate a chronic trajectory of health deterioration, underscoring the need for early identification and preventive action.

Screening for OSA using accessible tools and evaluating observable anatomical signs during routine dental examinations has emerged as a strategic approach to improve referral processes, facilitate early diagnosis, and prevent long-term consequences, ultimately enhancing patients’ quality of life [7,8]. Dental professionals, due to their routine and detailed examination of the orofacial region, are uniquely positioned to detect anatomical markers suggestive of OSA, such as hypertrophic palatine tonsils, high Mallampati scores, or specific craniofacial patterns [9,10,11]. When these findings are integrated with structured questionnaires such as the STOP-BANG or the Epworth Sleepiness Scale, the predictive value of clinical screening may be significantly enhanced [12].

Among university students, OSA often remains undiagnosed due to the subtlety or non-specificity of symptoms and the lack of standardized screening protocols in academic or primary care settings. The early identification of anatomical and functional risk markers is essential to address this public health issue in a vulnerable but frequently overlooked population [13,14]. Furthermore, limited data on OSA prevalence and clinical profiles in Latin American university populations highlight the need for region-specific epidemiological evidence to support culturally and structurally appropriate interventions [15,16].

This study addresses this gap by conducting a multidimensional assessment of OSA risk among young adults, integrating validated clinical tools such as the STOP-BANG questionnaire and the Epworth Sleepiness Scale, along with anatomical evaluations including the Mallampati index, palatine tonsil classification, facial profile, and Angle’s molar classification. Anthropometric parameters, including body mass index (BMI) and neck circumference, were also considered given their established association with OSA [17,18,19,20,21,22].

Accordingly, the main objective of this study is to identify anatomical and clinical predictors of moderate to high OSA risk among university students, with particular emphasis on sex-based differences. Given the limited evidence in this population and the importance of early diagnosis, these findings aim to inform effective screening strategies and reinforce the interdisciplinary role of dental professionals in the early management of OSA.

## 2. Materials and Methods

### 2.1. Study Design

This study employed a cross-sectional, descriptive, and analytical design conducted in an urban university population of young Colombian adults. The study was developed in accordance with the STROBE guidelines for observational research [23]. The protocol was approved by the Ethics Committee of the Autonomous University of Manizales (Record 136; Approval date: 10 August 2022) and complies with Resolution No. 8430 of 1993 of the Colombian Ministry of Health, as well as with the ethical principles of the Declaration of Helsinki [24]. During the preparation of this manuscript, the authors used ChatGTP-4-turbo to improve gram-matical style. The authors have reviewed and edited the output and take full responsibility for the content of this publication.

### 2.2. Context

Sleep-disordered breathing, particularly obstructive sleep apnea (OSA), represents a public health issue with a significant impact on quality of life and overall well-being [25]. Early identification of anatomical, functional, and clinical risk factors is essential for timely diagnosis and management. In young university populations, where prevalence and clinical expression may differ from older age groups, it is crucial to rely on validated tools and standardized protocols that allow a comprehensive and reliable assessment. This approach enables the examination of relationships between orofacial anatomical features, daytime sleepiness, and anthropometric parameters as a multidimensional framework to better understand the etiology and associated risks of OSA.

### 2.3. Participants

The sample consisted of 340 university students aged between 18 and 28 years, selected from a finite population of 2770 individuals enrolled at an urban university in Manizales, Colombia. Simple random sampling was used to ensure representativeness and diversity in terms of sex and socioeconomic status. Inclusion criteria included affiliation with a Health Service Provider and the ability to understand and adequately respond to the instruments in Spanish.

Exclusion criteria included pregnancy, previous diagnosis of OSA, or the presence of chronic cardiovascular, respiratory, or neurological diseases that could affect respiratory function or bias study outcomes. Although tools such as STOP-BANG and ESS are designed to be broadly applicable, individuals with chronic systemic diseases may present overlapping symptoms—such as fatigue, dyspnea, or poor sleep quality—that could confound the accurate assessment of OSA-related risk in healthy populations. Therefore, these individuals were excluded to improve internal validity and reduce clinical heterogeneity in the sample.

Participants who habitually consumed tobacco, alcohol, or recreational drugs were also excluded, as chronic substance use has been associated with alterations in sleep architecture, increased risk of upper airway obstruction, and changes in neurocognitive perception of sleepiness. These factors could distort STOP-BANG and ESS scores independently of true OSA risk. Excluding these participants helped to control potential confounders related to substance-induced sleep disturbances.

Likewise, participants who had recently undergone procedures that could alter airway anatomy or function, such as orthognathic surgery or recent dental treatments, were excluded. All participants provided written informed consent prior to participation in accordance with ethical research standards.

### 2.4. Procedure

Each participant completed an examination form including sociodemographic information such as sex, age, address, phone number, and health insurance provider. They were then assessed using various validated instruments following standardized protocols, as detailed below, to ensure data accuracy and reproducibility.

All clinical and anatomical assessments, as well as the administration of the questionnaires (STOP-BANG and ESS), were conducted on the same day to ensure procedural consistency. A single trained and calibrated examiner performed all evaluations to minimize inter-observer variability.

#### 2.4.1. Socioeconomic Status

Socioeconomic classification was determined according to Colombia’s official stratification system, which categorizes the population into six strata based on residential and environmental characteristics. This system, implemented by the National Administrative Department of Statistics and local authorities, aims to support the allocation of subsidies and differentiated rates for public utilities. The strata are defined as follows [26]:

Stratum I: Areas with low socioeconomic and physical conditions, typically with substandard housing and limited access to basic services.

Stratum II: Similar to Stratum I, but with slightly better housing and service access.

Stratum III: Middle-low socioeconomic level with adequate housing and basic services.

Stratum IV: Middle socioeconomic level with well-structured housing and efficient access to public services.

Stratum V: Upper-middle level areas with high-quality housing and full services.

Stratum VI: The highest socioeconomic level, with luxury housing, infrastructure, and optimal services.

#### 2.4.2. Body Composition

Height was measured using a portable stadiometer (Cescorf, São Paulo, Brazil) with a maximum length of 300 cm, validated for anthropometric purposes by the International Society for the Advancement of Kinanthropometry (ISAK) [27]. Body weight was recorded using a SECA digital scale (model 803), which complies with ISAK recommendations for a range of 0 to 150 kg and a precision of 100 g [27]. Body mass index (BMI) was calculated using the formula [28,29]:

BMI = Weight (kg)/Height (m^2^)

BMI categories were based on the cut-off points recommended by the World Health Organization (WHO) [29]:

Underweight: BMI < 18.5 kg/m^2^

Normal weight: BMI 18.5–24.9 kg/m^2^

Overweight: BMI 25.0–29.9 kg/m^2^

Obesity: BMI > 30.0 kg/m^2^

Neck circumference was also measured using a metallic measuring tape (Cescorf) with 1 mm precision, positioned below the laryngeal prominence and perpendicular to the long axis of the neck. Risk thresholds were defined as >43 cm for men and >41 cm for women [29,30,31].

#### 2.4.3. OSA Screening

OSA risk was assessed using the STOP-BANG questionnaire [17], a validated screening tool in various populations. It consists of 8 dichotomous items (“Yes” or “No”): snoring, daytime fatigue, observed apnea, high blood pressure, BMI > 30 kg/m^2^, age > 50 years, neck circumference > 40 cm, and male sex. Each affirmative response scores 1 point, with a total score categorizing risk as low (0–2), moderate (3–4), or high (5–8).

The Spanish version of the questionnaire has demonstrated good reliability (Cronbach’s alpha = 0.767, r = 0.777) and acceptable validity (Kappa coefficient = 0.444). These metrics support their utility in clinical and population-based settings [17,32,33].

#### 2.4.4. Daytime Sleepiness

Daytime sleepiness was assessed using the Epworth Sleepiness Scale (ESS), which evaluates the likelihood of dozing off in eight daily situations, rated from 0 to 3 per item. The total score ranges from 0 to 24. A score of 0–9 is considered normal; 10–24 suggests the need for clinical evaluation. Specifically, scores of 11–15 suggest mild to moderate sleep apnea, while scores >16 are associated with severe OSA or narcolepsy [18].

The Spanish version has been validated in Colombian university students, showing high internal consistency (Cronbach’s alpha = 0.82), supporting its use as a reliable tool for detecting excessive daytime sleepiness in clinical and population-based settings [18,34,35].

#### 2.4.5. Anatomical Risk of Upper Airway Obstruction

Anatomical risk was evaluated using the Mallampati index, which classifies oropharyngeal visibility with the participant seated, mouth fully open, and no phonation. It is divided into four classes [19]:

Class I: Soft palate, fauces, uvula, and pillars visible

Class II: Soft palate, fauces, and uvula visible

Class III: Soft palate and base of uvula visible

Class IV: Only hard plate visible

This index has been widely used in clinical settings and has shown moderate validity in predicting upper airway obstruction, especially when combined with other tools such as STOP-BANG [36]. Validation studies report kappa concordance coefficients around 0.30, depending on examiner expertise, indicating moderate reliability in clinical practice [37].

#### 2.4.6. Palatine Tonsil Grading

Tonsil grading was conducted with the participant seated and mouth open at rest. Upon adequate visualization, the Friedman scale was used to evaluate palatine tonsil size based on oropharyngeal space occupation [38]:

Grade I: Up to 25%

Grade II: 25–50%

Grade III: 50–75%

Grade IV: Over 75%

This scale has demonstrated moderate reliability with an intraclass correlation coefficient (ICC) of 0.647 and a Cronbach’s alpha of 0.879, indicating strong internal consistency and acceptable interrater agreement [39].

#### 2.4.7. Occlusal Classification

Occlusal relationships were assessed using Angle’s classification, which evaluates the anteroposterior relationship between the first upper and lower molars, based on the position of the mesiobuccal cusp (MV) of the upper first molar relative to the mesiobuccal groove of the lower first molar [40]:

Class I: MV cusp of the upper molar aligns with the groove of the lower molar

Class II: MV cups are anterior to the groove

Class III: MV cusp is posterior to the groove

This system is widely used in orthodontics due to its simplicity and diagnostic utility, although its reliability can be affected by malocclusions or missing teeth [20,41].

#### 2.4.8. Facial Profile

Facial profile was assessed via photographic analysis using the angle formed by three anatomical landmarks: glabella (G), subnasale (Sn), and soft tissue pogonion (Pg). To ensure measurement accuracy, the subject’s head was positioned with the Frankfort plane (line from external auditory canal to infraorbital point) parallel to the floor and the head in a neutral position.

The resulting FSA angle (Frankfort plane–Subnasale–Soft-tissue Pogonion angle) was used to classify the facial profile into three categories [21]:

Straight: 165° to 175°

Convex (prognathic): <165°

Concave (retrognathic): >175°

This method has been validated as a reliable and non-invasive alternative to cephalometric radiographs, with good correlation in both radiographic and photographic assessments, especially in clinical and epidemiological settings without access to lateral X-rays [21].

### 2.5. Bias

Several limitations and potential sources of bias should be considered when interpreting the results. Although selection bias was minimized through probabilistic sampling and the use of validated instruments, voluntary participation may have introduced self-selection bias, potentially overrepresenting individuals with greater interest or awareness of the subject, which could inflate results. The sample predominantly included young individuals (17–28 years) from middle socioeconomic strata (2, 3, and 4), which may limit generalizability [42].

Self-reported or proxy-reported data, particularly for variables such as snoring, daytime sleepiness, and restfulness, may have introduced recall or reporting bias, affecting data accuracy [42]. Additionally, potential confounding variables such as alcohol consumption, tobacco use, or medication intake were not controlled for, possibly influencing outcomes and introducing confounding bias [43].

Finally, the study was conducted exclusively in an urban university setting in Manizales, Colombia, limiting extrapolation to rural or demographically different populations [44].

### 2.6. Sample Size

Sample size was calculated using the finite population correction (fpc) factor based on the following parameters: total population (N) of 2770 university students, expected proportion (p) of 50%, margin of error ±5% (d = 0.05), and design effect (EDFF) of 1.0 given the use of simple random sampling. The formula applied was:

n = [EDFF × N × p(1 − p)]/[(d^2^/Z^2^_1_ − α/2) × (N − 1) + p(1 − p)]

Based on this approach, the minimum required sample size for a 95% confidence level was 335 participants. The final sample of 340 participants met this requirement. This method aligns with standard methodological recommendations for finite populations, ensuring adequate statistical power and representativeness in psychometric health research, especially when sampling procedures preserve population heterogeneity [45,46,47].

### 2.7. Statistical Analysis

Data were analyzed using SPSS version 27.0 (IBM Corp., Armonk, NY, USA). Continuous variables were described using measures of central tendency (mean) and dispersion (standard deviation), while categorical variables were reported as frequencies and percentages. To assess sex-based differences, Student’s *t*-test for independent samples or the Mann–Whitney U test was used based on data normality, and chi-square tests were used for categorical variables. Effect sizes were calculated to estimate the magnitude of differences: Cohen’s d (0.1 = small, 0.3 = moderate, ≥0.7 = large) for continuous variables, and Cramer’s V (0.1 = small, 0.3 = moderate, ≥0.7 = large) for categorical variables [48,49,50].

To identify predictors of moderate/high OSA risk—defined as a STOP-BANG score ≥ 3—binary logistic regression models stratified by sex were conducted due to known anatomical and physiological differences. Each model included regression coefficients (B), odds ratios (OR), and their 95% confidence intervals (95% CI). For the male subgroup, the variables included were age, neck circumference, ESS score, Mallampati index, Angle’s molar classification, and facial profile. For females, Angle’s molar classification was excluded due to multicollinearity and model instability. Statistical significance was set at *p* < 0.05.

## 3. Results

Table 1 presents demographic, anthropometric, and clinical characteristics of the 340 participants (148 males and 192 females). Statistically significant sex differences were observed: males had higher age (*p* = 0.003), neck circumference (*p* < 0.001), and STOP-BANG scores (*p* < 0.0001), while females showed higher scores on the ESS (*p* < 0.001). Anatomical differences were also identified for the Mallampati index (*p* = 0.01), Angle classification (*p* = 0.04), and facial profile (*p* = 0.02), with a predominance of prognathism in men and retrognathism in women. The prevalence of moderate-to-high OSA risk (STOP-BANG ≥3) was significantly higher in men (23%) than in women (3.1%).

Table 2 shows the global logistic regression model. Neck circumference (OR = 0.57; *p* < 0.001) and daytime sleepiness (OR = 0.86; *p* = 0.01) were significantly associated with moderate/high OSA risk, while age showed a marginal trend toward significance.

Given anatomical and physiological sex differences, stratified models were applied. Among males, neck circumference (OR = 0.62; *p* < 0.001) and Angle’s classification (OR = 0.54; *p* = 0.04) were significant predictors (Table 3).

In the female model, Angle classification was excluded due to statistical instability. In this group, neck circumference (OR = 0.35; *p* = 0.01) and Epworth sleepiness score (OR = 0.60; *p* = 0.03) predicted OSA risk, while facial profile showed a borderline trend toward significance (*p* = 0.07) (Table 4).

## 4. Discussion

This cross-sectional study evaluated the association of anatomical, clinical, and physiological factors with OSA risk in a young university population, using the STOP-BANG questionnaire as the primary screening tool. Our findings indicate that male sex is associated with a higher prevalence of moderate-to-high OSA risk, and that neck circumference and daytime sleepiness were the most consistent predictors. These findings are consistent with the known multifactorial pathophysiology of OSA, which integrates structural and neuromuscular determinants, along with demographic and clinical features that modulate individual susceptibility. The use of validated tools such as the STOP-BANG and ESS enables the identification of individuals at increased risk, particularly in populations where polysomnography is not routinely accessible.

Significant sex-based differences were observed in multiple variables. Men were older, had larger neck circumference, and presented higher STOP-BANG scores, which aligns with previous studies identifying male sex as an independent OSA risk factor [51,52,53]. These differences likely reflect underlying biological mechanisms, including greater upper airway collapsibility, reduced pharyngeal dilator muscle responsiveness, and differences in fat distribution, with men tending to accumulate more adipose tissue in the cervical and abdominal regions, which directly impacts airway patency during sleep. Additionally, the influence of testosterone has been implicated in reduced ventilatory drive and greater susceptibility to upper airway obstruction in men, further explaining the elevated risk scores.

Conversely, women reported higher levels of daytime sleepiness on the Epworth Scale. This may reflect differences in symptom perception or expression; notably, some individuals with severe OSA may not exhibit excessive daytime sleepiness [54]. Indeed, women with OSA are more likely to report non-classical symptoms, including fatigue, insomnia, morning headaches, or depressive mood, rather than the hallmark features of snoring and witnessed apneas, which are more commonly reported in men. This sex-specific symptomatology complicates clinical recognition and may result in underdiagnosis among women. Nevertheless, this result should be interpreted with caution, given inconsistencies reported in the literature regarding the ESS [55,56]. Specifically, the ESS has been shown to have variable sensitivity and specificity across populations, and its subjective nature may be influenced by cultural, psychosocial, or behavioral factors. In young adults, where sleep patterns may already be irregular due to academic or social demands, daytime sleepiness may not always correlate with the presence or severity of OSA, necessitating cautious interpretation of ESS scores in isolation.

From an anatomical perspective, we found significant sex differences in the Mallampati index, Angle’s classification, and facial profile—prognathism being more common in males, and retrognathism in females. These structural variations could be associated with differential susceptibility to upper airway obstruction [57,58]. For instance, retrognathism has been linked to posterior displacement of the tongue and soft palate, increasing the likelihood of pharyngeal collapse, while prognathism may provide more anterior support to the airway. Such findings highlight the role of craniofacial architecture in modulating OSA risk and support the integration of orofacial assessments in screening protocols. The observation of increased retrognathism in females is particularly relevant, as this skeletal pattern may exacerbate upper airway collapsibility, especially when combined with other risk factors such as obesity or high Mallampati scores.

The Frankfort angle was more frequently convex in women; a pattern previously associated with higher risk of pharyngeal collapse and subsequent apnea or hypopnea events during sleep [59,60,61,62]. A convex facial profile often indicates retrusive mandibles and/or maxillary protrusion, both of which can alter the spatial dynamics of the upper airway. These cephalometric features have been associated with a narrower retropalatal space and decreased airway stability during the hypotonic state of sleep. In the context of young adults, identifying such patterns may provide early markers of risk and support the need for orthodontic or myofunctional interventions in preventive strategies.

The global logistic model indicated that neck circumference and ESS scores were independent predictors of moderate/high OSA risk. Neck circumference has consistently been validated as a reliable anatomical marker for upper airway obstruction, which our results further support [63,64]. Its utility lies in its simplicity and its strong correlation with peripharyngeal fat deposition and soft tissue crowding. In our study, this parameter retained predictive value even after adjusting for other anatomical and demographic factors, underscoring its central role in OSA pathogenesis. Interestingly, age exhibited a marginal association, possibly reflecting the youth of our sample and the low number of high-risk cases. This is not unexpected, as younger adults may have fewer structural or metabolic risk factors than older populations, and age-related changes in airway tone and neuromuscular compensation may not yet be prominent. However, the inclusion of age in screening models remains important, given its established predictive value across broader age ranges.

Sex-stratified models revealed distinct patterns. Among males, neck circumference and molar Angle classification were significant predictors, suggesting that mandibular morphology may be particularly relevant in this group [65,66]. Class II malocclusion, which implies mandibular retrusion, has been linked to diminished airway space and increased vulnerability to obstruction during supine sleep. In young men, who may not yet exhibit other systemic risk factors such as hypertension or metabolic syndrome, the role of craniofacial anatomy becomes particularly salient. These findings reinforce the value of integrating dental and orthodontic assessments into early OSA risk screening, especially in male populations with suggestive anatomical features.

In women, neck circumference and daytime sleepiness were significantly associated with risk, while facial profile showed a near-significant trend. These results suggest that in females, subjective clinical symptoms such as sleepiness may have greater predictive value than classical anatomical indicators. This is consistent with studies describing a less typical clinical presentation of OSA in women, who more often report nonspecific symptoms such as insomnia, fatigue, and daytime sleepiness, rather than classic signs such as snoring or observed apneas [67,68]. The complex interplay of hormonal influences, anatomical variations, and sociocultural factors may modulate how OSA manifests in women and may necessitate sex-specific diagnostic approaches. Furthermore, general or abdominal obesity in young adults appears to be more strongly linked to OSA risk when clinical and behavioral cofactors are considered [69]. While BMI alone may not capture fat distribution patterns adequately, neck circumference serves as a more direct marker of central adiposity and potential upper airway compromise. The non-significance of Angle classification in women may be due to statistical instability related to the low number of high-risk cases, limiting the power to detect associations. Nevertheless, the observed trends warrant further investigation in larger, sex-stratified cohorts that explore the interaction between craniofacial morphology and clinical symptomatology in female OSA phenotypes.

## 5. Strengths and Limitations

This study has several methodological strengths. First, the large sample size (n = 340) allowed for robust sex-based comparative analysis and adequate statistical power in regression modeling. The use of validated instruments (STOP-BANG and ESS) supports the reliability of OSA risk assessment. Additionally, the inclusion of clinically accessible anatomical parameters (Mallampati index, Angle classification, facial profile) provides an integrative approach from both dental and medical perspectives, enhancing the applicability of the findings in primary and specialized care settings.

However, this study also presents some important limitations. The cross-sectional design prevents establishing causal relationships, limiting temporal inference between factors and OSA risk. OSA risk was assessed through a screening tool (STOP-BANG) without polysomnographic confirmation, which could lead to false positives or negatives. Although this limitation is common in epidemiological research, future studies should consider the use of home sleep apnea testing devices—such as WatchPAT or ApneaLink—that have been validated as alternatives to in-lab polysomnography and may improve diagnostic accuracy in population-based settings [70,71].

The sample comprised university students from Manizales (Colombia), which restricts generalizability to other populations. Furthermore, some anatomical variables were clinically assessed without imaging support, potentially introducing inter-observer variability.

## 6. Clinical Implications

The findings underscore the importance of early identification of OSA risk factors in young adults—a population often underestimated in clinical practice. Neck circumference and daytime sleepiness, both simple and non-invasive measures, emerged as relevant predictors of moderate/high OSA risk even among young, apparently healthy individuals. Significant sex-based differences in anatomical and clinical factors suggest that OSA risk assessment should be sex-specific.

Implementing the STOP-BANG questionnaire in university or primary care settings may facilitate early screening and guide referrals for specialized evaluation, especially in men with Class III craniofacial features or increased neck circumference, and in women with persistent daytime sleepiness. These results support the use of low-cost, easily applicable tools as part of integrated preventive strategies to reduce OSA underdiagnosis in young populations.

## 7. Conclusions

The present study demonstrates that neck circumference and self-reported daytime sleepiness are significant predictors of moderate-to-high OSA risk in young adults, with important sex-based differences. Among men, molar Angle classification also predicted risk, while in women, ESS scores and facial profile showed greater predictive relevance. These associations highlight the importance of sex-specific anatomical and clinical variables in OSA risk assessment. Early screening using easily measurable parameters in dental or primary care settings can aid in identifying at-risk individuals. However, longitudinal studies and polysomnographic confirmation are needed to validate these findings and clarify their clinical implications. Overall, this study supports a multidisciplinary approach to OSA risk in young adults, promoting integrated preventive strategies involving both medicine and dentistry.

## Figures and Tables

**Table 1:** Description of the sample by sex.

**Continuous Variables**	**Total (n = 340)**	**Man (n = 148)**	**Women (n = 192)**	***p*** **-Value**	**Effect Size**
Age (years)	21.36 ± 2.37	21.79 ± 2.45	21.04 ± 2.25	0.003 *	0.32
BMI (kg/m^2^)	23.80 ± 3.91	23.82 ± 3.58	23.78 ± 4.15	0.94	0.01
Neck circumference (cm)	35.10 ± 4.43	38.18 ± 3.20	32.72 ± 3.74	<0.001 *	1.57
Epworth Sleepiness Scale	9.31 ± 4.31	8.16 ± 3.91	10.20 ± 4.41	<0.001 *	0.49
**Categorical Variables**	**Total (n = 340)**	**Man (n = 148)**	**Women (n = 192)**	***p*-Value**	**Effect Size**
Socioeconomic stratum I	13 (3.8%)	4 (2.7%)	9 (4.7%)	0.45	0.12
Socioeconomic stratum II	50 (14.7%)	19 (12.8%)	31 (16.1%)		
Socioeconomic stratum III	163 (47.9%)	79 (53.4%)	84 (43.8%)		
Socioeconomic stratum IV	66 (47.9%)	26 (17.6%)	40 (20.8%)		
Socioeconomic stratum V	27 (7.9%)	13 (8.8%)	14 (7.3%)		
Socioeconomic stratum VI	21 (6.2%)	7 (4.7%)	14 (7.3%)		
BMI. Low weight	17 (5%)	8 (5.4%)	9 (4.7%)	0.37	0.09
BMI: Normal weight	209 (61.5%)	88 (59.5%)	121 (63%)		
BMI: Overweight	91 (26.8%)	45 (30.4%)	46 (24%)		
BMI: Obesity	23 (6.8%)	7 (4.7%)	16 (8.3%)		
Stop-BANG: Low risk	300 (88.2%)	114 (77%)	186 (96.9%)	<0.0001 *	0.31
Stop-BANG: Moderate risk	38 (11.2%)	32 (21.6%)	6 (3.1%)		
Stop-BANG: High risk	2 (0.6%)	2 (1.4%)	0 (0%)		
Mallampati index: Class I	74 (21.5%)	22 (14.9%)	52 (27.1%)	0.01 *	0.17
Mallampati index: Class II	129 (38%)	55 (37.2%)	74 (38.5%)		
Mallampati index: Class III	81 (23.8%)	45 (30.4%)	36 (18.8%)		
Mallampati index: Class IV	56 (16.5%)	26 (17.6%)	30 (15.6%)		
Friedman Scale: Grade I	129 (38%)	53 (35.8%)	76 (39.6%)	0.79	0.05
Friedman Scale: Grade II	131 (38.5%)	61 (41.2%)	70 (36.5%)		
Friedman Scale: Grade III	72 (21.2%)	30 (20.3%)	42 (21.9%)		
Friedman Scale: Grade IV	8 (2.3%)	4 (2.7%)	4 (2.1%)		
Angle system: Class I	184 (54.1%)	77 (52%)	107 (55.7%)	0.04 *	0.14
Angle system: Class II	78 (22.9%)	28 (18.9%)	50 (26%)		
Angle system: Class III	78 (22.9%)	43 (29.1%)	35 (18.2%)		
Frankfort Angle: concave	47 (13.8%)	29 (19.6%)	18 (9.4%)	0.02 *	0.15
Frankfort Angle: Straight	174 (51.2%)	70 (47.3%)	104 (54.2%)		
Frankfort Angle: convex	119 (35%)	49 (33.1%)	70 (36.4%)		
Use medications	95 (27.9%)	36 (24.3%)	59 (30.7%)	0.24	0.07
No medications	245 (72.1%)	112 (75.7%)	133 (69.3%)		

*: statistical significance.

**Table 2:** Predictors of Moderate/High OSA Risk (STOP-BANG ≥ 3).

Predictor	B	OR	95% CI	*p*-Value
Age (years)	–0.17	0.84	0.71–1	0.05
Neck circumference (cm)	–0.56	0.57	0.48–0.68	<0.001 *
Epworth Sleepiness Scale	–0.15	0.86	0.77–0.96	0.01 *
Mallampati score	0.10	1.10	0.68–1.79	0.69
Molar Angle Classification	–0.49	0.61	0.35–1.06	0.08
Facial profile	0.52	1.68	0.97–2.92	0.07

*: statistical significance.

**Table 3:** Predictors of Moderate/High OSA Risk (STOP-BANG ≥ 3) in Men.

Predictor	B	OR	95% CI	*p*-Value
Age (years)	−0.12	0.89	0.73–1.08	0.24
Neck circumference (cm)	−0.48	0.62	0.51–0.76	<0.001 *
Epworth Sleepiness Scale	−0.12	0.89	0.79–1.01	0.06
Mallampati score	0.17	1.18	0.69–2.03	0.55
Molar Angle Classification	−0.62	0.54	0.30–0.97	0.04
Facial profile	1.81	6.12	0.84–44.69	0.07

*: statistical significance.

**Table 4:** Predictors of Moderate/High OSA Risk (STOP-BANG ≥ 3) in Women.

Predictor	B	OR	95% CI	*p*-Value
Age (years)	−0.23	0.80	0.56–1.13	0.20
Neck circumference (cm)	−1.05	0.35	0.16–0.75	0.01 *
Epworth Sleepiness Scale	−0.50	0.60	0.39–0.95	0.03 *
Mallampati score	−0.39	0.68	0.14–3.25	0.62
Facial profile	1.81	6.12	0.84–44.69	0.07

*: statistical significance.

## Data Availability

The data from this article will be made available by the authors on reasonable request.
